# The Impact of Reduced-Volume, Intensity-Modulated Radiation Therapy on Disease Control in Nasopharyngeal Carcinoma

**DOI:** 10.1371/journal.pone.0125283

**Published:** 2015-04-28

**Authors:** Yu-Wei Lin, Chia-Chun Chen, Li-Ching Lin, Steve P. Lee

**Affiliations:** 1 Department of Radiation Oncology, Chi Mei Medical Center, Tainan, Taiwan; 2 Institute of Biomedical Sciences, National Sun Yat-sen University, Kaohsiung, Taiwan; 3 The School of Medicine, Kaohsiung Medical University, Kaohsiung, Taiwan; 4 Department of Radiation Oncology, Liouying campus, Chi Mei Medical Center, Tainan, Taiwan; 5 School of Medicine, Taipei Medical University, Taipei, Taiwan; 6 Department of Radiation Oncology, David Geffen School of Medicine, University of California Los Angeles, Los Angeles, CA, United States of America; 7 Department of Radiation Oncology, VA Greater Los Angeles Healthcare System, Los Angeles, CA, United States of America; Ludwig-Maximilians University, GERMANY

## Abstract

**Objective:**

To investigate the feasibility of using intensity-modulated radiotherapy (IMRT) with reduced, high-dose target volumes for nasopharyngeal carcinoma (NPC).

**Methods:**

The first 57 patients (admitted from October 2005 to May 2008) were treated with large-target-volume IMRT (LV-IMRT). For the LV-IMRT group, the CTV at 70 Gy (CTV70) was delineated as the gross target volume (GTV) plus 7mm, with or without the first-echelon lymph-node region. The next 56 patients (admitted from June 2008 to November 2011) were treated with reduced-target-volume IMRT (RV-IMRT). For the RV-IMRT group, the CTV70 was delineated as the GTV alone.

**Results:**

The 4-year local recurrence-free, regional recurrence-free, distant metastasis-free, progression-free, and overall survival rates were 77.2%, 80.1%, 83.2%, 61.2%, and 74.4% for the LV-IMRT group and 83.5%, 92.6%, 89.1%, 78.5, and 91.0% for the RV-IMRT group, respectively. Late toxicity scoring of xerostomia was lesser in the RV-IMRT group than the LV-IMRT group (P < 0.001).

**Conclusions:**

The use of RV-IMRT for the treatment of NPC did not negatively affect survival rates but did reduce the late xerostomia events compared to LV-IMRT.

## Introduction

Controlling nasopharyngeal carcinoma (NPC) has been a challenge for radiation oncologists, particularly in endemic region. The locoregional control of NPC depends on high-dose radiation therapy; however the nasopharyngeal region is surrounding by critical, dose-limiting normal tissue. Therefore, a major breakthrough in NPC treatment has been the development of intensity-modulated radiation therapy (IMRT), and encouraging clinical results involving the use of IMRT to treat NPC have been reported from studies involving single institutions[1–5] as well as from clinical studies performed at multiple centers[6, 7].

Today, the local control rate of NPC is greater than 85%, with relatively few marginal failures being reported. However, the use of high-dose radiation in tissues adjacent to the clinical target volumes (CTVs) for the treatment of potentially subclinical disease is controversial[8–10]. In particular, increased high-dose treatment volumes inevitably increase the probability of both acute and late adverse effects. But, preserving excessive normal tissue, particularly in the parotid gland, can lead to unexpected failures[11]. The purpose of the present study was to investigate the feasibility and efficacy of using IMRT with reduced, high-dose CTVs and reduced, high-dose planning target volumes (PTVs) for the treatment of NPC.

## Methods and Materials

### Ethics Statement

This study was approved by the Institutional Review Board (IRB) at Chi Mei Medical Center (IRB No.10310-007). The institutional review board waived the need for written informed consent from the participants because this was a retrospective chart-review study.

### Patient selection

Between October 2005 and November 2011, 117 consecutive patients with nonmetastatic NPC were treated with definitive IMRT. Two patients were referred from another hospital, which delayed radiotherapy administration, and two other patients did not complete the planned radiotherapy course were excluded. Therefore, 113 patients in total were retrospectively analyzed. The pretreatment staging evaluation consisted of a physical examination, an endoscopic examination, chest radiography, computed tomography (CT) scanning/magnetic resonance imaging (MRI) of the head and neck, a bone scan, ultrasonography of the abdomen, and a dental assessment. Positron emission tomography (PET) and CT scans of the chest/abdomen were performed when clinically required. The tumors were staged according to the American Joint Committee on Cancer (AJCC) 1997 cancer-staging classification.

### Radiotherapy

#### Target delineation

**Fig 1:**
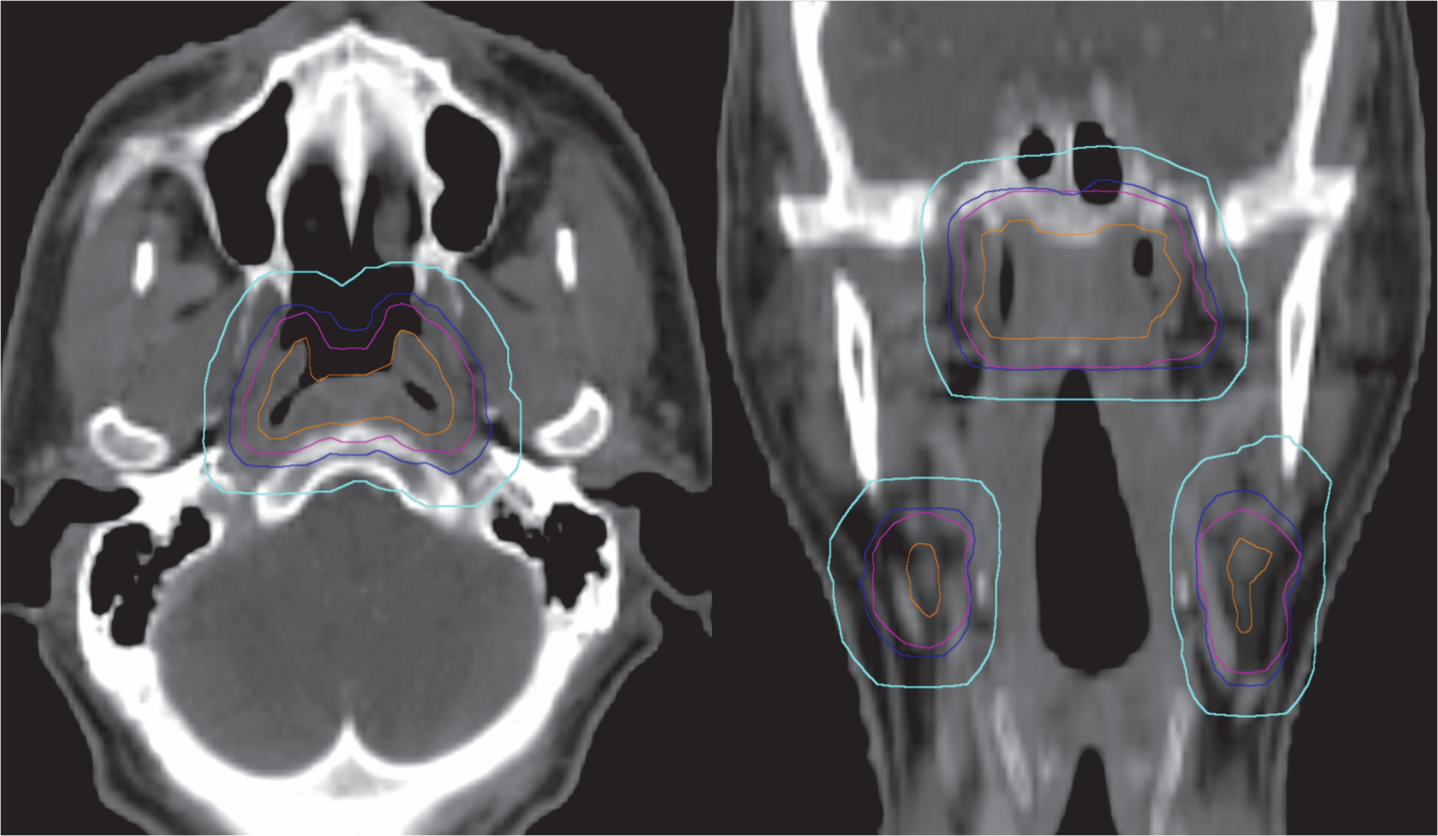
Illusion of the target delineations in the large-target-volume IMRT (LV-IMRT) and the reduced-target-volume IMRT (RV-IMRT). For the LV-IMRT group, the CTV at 70 Gy (CTV70, blue line) was delineated as the gross target volume (GTV, orange line) plus 7mm. The PTVs (cyan line) were constructed by expanding the CTVs by 7 mm. For the RV-IMRT group, the CTV70 was delineated as the GTV alone (orange line). The PTVs (magenta line) were expanded 5mm from the CTVs.

During October 2005 to May 2008, the first 57 enrolled patients were treated with large-target-volume IMRT (LV-IMRT). The gross tumor volume (GTV) included the primary nasopharyngeal tumor and the affected lymph nodes, as determined by clinical, endoscopic, and radiologic examinations. For the LV-IMRT group, the clinical target volume of 70 Gy (CTV70) was delineated as the GTV plus 7mm[12, 13] (Fig 1), with or without the first-echelon lymph-node region, according to the discretion of the physician. The first-echelon lymph-node region was defined as the retropharyngeal region of the primary nasopharyngeal tumor and the subdigastric region of the gross lymph nodes of the upper neck region[14]. The CTV63 covered the CTV70 as well as the high-risk regions[15], including the parapharyngeal spaces, the posterior third of the nasal cavities and maxillary sinuses, the pterygoid processes, the base of the skull, the lower half of the sphenoid sinus, the anterior one third of the clivus, and the lymphatic regions, including the node-positive neck region, the bilateral retropharyngeal nodes, level II, III, and VA. The CTV56 covered the low-risk regions, including the remaining level IV-VB and the uninvolved neck regions. The PTVs were constructed by expanding the corresponding CTVs by 7 mm.

The reduced-target-volume IMRT (RV-IMRT) was applied to the 56 patients who were enrolled after June 2008. For the RV-IMRT group, the CTV70 was delineated as the GTV without any expansion (Fig 1). The CTV63 included the CTV70 and the high-risk regions, and the CTV56 covered the low-risk regions, as described for LV-IMRT. The PTVs were expanded by 5mm from the corresponding CTVs.

For LV-IMRT and RV-IMRT, in some cases, the expanded margin of the CTV70 to PTV70 was less than 5mm to 7mm if the CTV70 was close to critical neural tissue, such as the brain stem, spinal cord, or optic apparatus.

#### Radiation doses and techniques

The dose prescription was 70 Gy for the PTV70 in 35 fractions, 63Gy for the PTV63 in 35 fractions, and 56Gy for the PTV56 in 35 fractions. Treatment was delivered daily, 5 days a week. Full-length, simultaneous-boost IMRT was given to all NPC patients. Treatment plans were generated using the Eclipse treatment planning system (Varian Medical Systems) and the Pinnacle treatment planning system (Philips Medical Systems, Highland Heights, OH). Normal tissues were contoured and constrained. When considering acceptable treatment volumes, first priority was given to the protection of critical neurologic structures, followed by target volume coverage, and finally to the avoidance of other normal tissues. IMRT was delivered with 6-MV X-ray beams modulated using Clinical iX (Varian Medical Systems, Palo Alto, CA) and Precise (Elekta Oncology, Stockholm, Sweden) coming from typically 7 coplanar angles; additional angles were applied when necessary.

### Chemotherapy

Patients from both groups with diseases of stage T2b or higher or with N+ disease also received concurrent chemotherapy. Cisplatin (30–40 mg/m^2^) was administered weekly at the same time as the radiotherapy, followed by adjuvant chemotherapy involving four courses of monthly Cisplatin (80mg/m^2^) and 5-fluorouracil (1000mg/m^2^).

### Patient evaluation

Patient follow-ups were performed regularly over the course of the first year on the first, third, sixth, ninth, and twelfth months post-operation. After the first year, follow-ups took place every 3 months for the second and third years and every 6 months for the fourth and fifth years; annual follow-up after the fifth year. CT or MRI scans were performed 2–4 months after radiotherapy or when clinically indicated to assess the treatment response.

### Definition of failure patterns and adverse effects

Local failure was defined as recurrence within the nasopharyngeal region. Regional failure was defined as failure within the regional lymphatic drainage area, including the retropharyngeal region and the bilateral neck region distinct from the primary site. Locoregional failure was defined as either local failure or regional failure. Distant metastasis was defined as a recurrent lesion outside the head and neck region. Progression-free survival was defined as the absence of failure or recurrence within local, regional, and distant regions. Local or regional failure and distant metastasis were confirmed using various methods, including physical examination, tissue biopsy, surgical intervention, and serial imaging. In-field failure was defined as 95% of the failure volume within 66.5Gy (95% of 70Gy). Marginal failure was defined as 20–95% of the failure volume within 66.5Gy. Out-field failure was determined as <20% of the failure volume within 66.5Gy. The acute adverse effects mucositis and dermatitis and the late adverse effect xerostomia were scored according to the Common Toxicity Criteria Adverse Effect (CTCAE v3.0) system.

### Statistical methods

Overall survival (OS), progression-free survival (PFS), distant metastasis-free survival (DMFS), local recurrence-free survival (LRFS), regional recurrence-free survival (RRFS), and locoregional recurrence-free survival (LRRFS) were analyzed as the endpoints in the two groups. The differences between the proportions of the clinical characteristics in each group were evaluated using Fisher’s exact test. The dosimetric characteristics for the critical structures were compared using a Student’s *t* test. The relevance of the clinical factors to various failures was analyzed using univariate and multivariate analyses with the Cox proportional hazards model. Estimates for OS, PFS, DMFS, LRFS, RRFS, and LRRFS were calculated using the Kaplan-Meier method, and comparisons between groups were performed using log-rank tests. All tests were two-tailed, and a probability value of <0.05 was considered statistically significant.

## Results

### Patient clinical characteristics

The median follow-up time was 66 months (range, 3–111 months) for the entire patient population and 84 (range, 3–111 months) and 57 (range, 7–79 months) months for the LV-IMRT and RV- IMRT groups, respectively. Table 1 summarizes the patient clinical characteristics. The median ages in the LV-IMRT and RV-IMRT groups were 50 and 49 years of age, respectively. The most common histological type was World Health Organization Type IIb (LV-IMRT, 70%; RV-IMRT, 63%). The majority of the patients presented with locally advanced diseases of stage III or IV, which comprised 80% and 78% of the patients in the LV-IMRT and RV-IMRT groups, respectively. None of the clinical characteristics listed in Table 1 were significantly different between the two treatment groups.

**Table 1:** Patient clinical characteristics.

	LV-IMRT	RV-IMRT	P value
Patient No.	57	56	
Age (median)	50	49	0.813
>60	12 (21%)	10 (18%)	
< 60	45 (79%)	46 (82%)	
Gender			0.196
M	46 (81%)	39 (70%)	
F	11 (19%)	17 (30%)	
WHO classification			0.319
I	1 (2%)	4 (8%)	
IIa	16 (28%)	15 (29%)	
IIb	40 (70%)	32 (63%)	
T stage			0.404
1	8 (14%)	14 (25%)	
2	32 (56%)	24 (43%)	
3	3 (5%)	4 (7%)	
4	14 (25%)	14 (25%)	
N stage			0.505
0	10 (18%)	6 (11%)	
1	15 (26%)	11 (20%)	
2	28 (49%)	33 (59%)	
3	4 (7%)	6 (11%)	
Stage			0.733
1	1 (2%)	4 (7%)	
2	10 (18%)	8 (14%)	
3	29 (50%)	27 (48%)	
4a	13 (23%)	12 (21%)	
4b	4 (7%)	5 (9%)	
Chemotherapy			1.000
without	5 (9%)	5 (9%)	
with	52 (91%)	51 (91%)	

LV-IMRT, large-target-volume intensity-modulated radiotherapy; RV-IMRT, reduced-target-volume intensity-modulated radiotherapy; SEM., standard error mean; *, Statistical significance.

### Dosimetric characteristics

Table 2 summarizes the dosimetric data. The mean volumes of the GTVs in the LV-IMRT and RV-IMRT groups were 36.1 and 51.1 ml, respectively. No statistically significant differences in volume were observed for the GTVs between the LV-IMRT and RV-IMRT groups (P = 0.058). The mean values for CTV70, PTV, and 70Gy volume in the LV-IMRT group were 258.2ml, 482.4ml, and 556.1ml, respectively. The mean values for CTV70, PTV, and 70Gy volume in the RV-IMRT group were 51.1ml, 182.9ml, and 225.8ml, respectively. Significantly lower values for CTV70, PTV, and 70Gy volume were observed in the RV-IMRT group compared to the LV-IMRT group. The mean dose delivered to the parotid gland and the percentage volume of the parotid gland receiving 30Gy (V30) were both significantly lower in the RV-IMRT group compared to the LV-IMRT group for both the right-side parotid glands.

**Table 2:** Patient dosimetric characteristics.

	LV-IMRT	RV-IMRT	P value
GTV volume (mean, ml)(SEM)	36.1 (4.1)	51.1 (6.6)	0.058
CTV_70_ volume (mean, ml)(SEM)	258.2 (23.1)	51.1 (6.6)	<0.0001*
PTV_70_ volume (mean, ml)(SEM)	482.4 (37.5)	182.9 (21.3)	<0.0001*
Volume of 70Gy (mean, ml)(SEM)	556.1 (139.1)	225.8 (22.2)	0.023*
GTV coverage (mean, %)(SEM)	94.4(1.7)	98.1 (0.6)	0.045*
PTV_70_ coverage (mean, %)(SEM)	84.1(1.0)	93.6 (0.6)	<0.0001*
Mean parotid gland dose			
Right (mean, Gy)(SEM)	40.5 (117.7)	36.7 (81.2)	0.010*
Left (mean, Gy)(SEM)	39.6 (98.7)	37.6 (67.0)	0.105
V30 of the Parotid gland			
Right (mean, %)(SEM)	56.9 (1.8)	51.6 (1.6)	0.029*
Left (mean, %)(SEM)	58.1 (2.0)	54.3 (1.8)	0.152

LV-IMRT, large-target-volume intensity-modulated radiotherapy; RV-IMRT, reduced-target-volume intensity-modulated radiotherapy; SEM., standard error mean

*, Statistical significance.

### Failure patterns

#### Survival analysis

**Fig 2:**
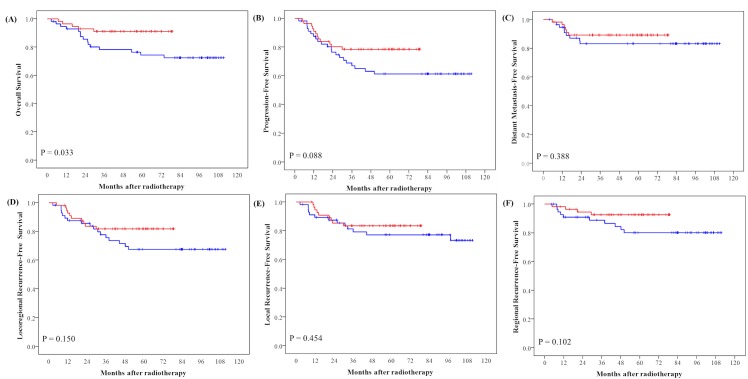
Kaplan-Meier curves illustrate the survival of two-group patients who underwent radiotherapy. Large-target-volume intensity-modulated radiotherapy [red line] and reduced-target-volume intensity-modulated radiotherapy [blue line]), including (A) overall survival, (B) progression-free survival, (C) distant metastasis-free survival, (D) locoregional recurrence-free survival, (E) local recurrence-free survival, (F) regional recurrence-free survival.

Fig 2 shows the survival outcomes for the LV-IMRT and RV-IMRT groups. For the LV-IMRT group, the 4-year OS, PFS, DMFS, LRFS, RRFS, and LRRFS rates were 74.4%, 61.2%, 83.2, 77.2%, 80.1%, and 67.5%, respectively. For the RV-IMRT group, the 4-year OS, PFS, DMFS, LRFS, RRFS, and LRRFS rates were 91.0%, 78.5%, 89.1%, 83.5%, 92.6%, and 81.7%, respectively. There were no statistically significant differences in the failure rates between the two groups.

#### Failure patterns

Failure patterns are summarized in Table 3. Seventeen cases (29.8%) of locoregional failure occurred in the LV-IMRT group, and ten cases (17.9%) occurred in the RV-IMRT group. The most common failure site in both groups was at the primary tumor site within the nasopharynx (NP). Twelve cases showed NP failure in the LV-IMRT group and nine cases showed NP failure in the RV-IMRT group. The majority of the cases of NP failure in both groups involved T4 cases (8 patients among 12 NP failures in the LV-IMRT group; 5 patient among 9 NP failures in the RT-IMRT group).

**Table 3:** Failure patterns after radiotherapy.

	LV-IMRT	RV-IMRT
	Cases	Cases
Locoregional failure (%)	17 (29.8%)	10 (17.9%)
Failures at NP only (%)	7 (12.3%)	6 (10.7%)
Failures at NP and original LN regions (%)	5 (8.8%)	3 (5.4%)
Failures at original LN regions (%)	1 (1.8%)	1 (1.8%)
Failure at new LN regions (%)	4 (7.0%)	0 (0.0%)
Distant metastasis (%)	9 (15.8%)	6 (5.6%)
In-field failures (%)	12 (21.1%)	9 (16.1%)
Marginal failures (%)	2 (3.5%)	0 (0.0%)
Out-field failures (%)	3 (5.3%)	0 (0.0%)
Total failures at NP (T4,cases)	12 (T4, 8)	9 (T4, 5)

LV-IMRT, large-target-volume intensity-modulated radiotherapy; RV-IMRT, reduced-target-volume intensity-modulated radiotherapy; Sig., significance

*, Statistical significance.

Among the all 27 locoregional-failure cases, the majority were in-field failures (21 cases, 77.8%; 12 in the LV-IMRT group and 9 in the RV-IMRT group). Two marginal failures and three out-field failures were also observed in the LV-IMRT group. No marginal or out-field failures occurred in the RV-IMRT group.

### Risk factors for disease control

Multivariate Cox regression analysis of survival rates (Table 4) showed that disease treatment was not adversely affected by reduced-volume IMRT. T stage was found to be an important prognostic factor for NPC disease control. In particular, the T4 disease status showed the highest hazard ratio (HR) for OS, PFS, DMFS, and LRRFS (HR, 10.53, 8.49, 10.55, and 12.59, respectively). Grouping also demonstrated as an independent prognostic role in PFS and LRRFS.

**Table 4:** Impact of the prognostic factors on the treatment results based on multivariate Cox regression analysis.

Variable	Category	OS			PFS			DMFS			LRRFS		
		RR	95% CI	P value	RR	95% CI	P value	RR	95% CI	P value	RR	95% CI	P value
Group	LV-IMRT	1		0.054	1		0.033*	1		0.132	1		0.035*
	RV-IMRT	0.35	0.12–1.02		0.45	0.21–0.94		0.43	0.14–1.30		0.41	0.18–0.94	
T stage	T1	1		0.032*	1		<0.0001*	1		0.003*	1		<0.0001*
	T2	3.19	0.40–25.4		1.43	0.40–5.11		1.28	0.14–11.67		1.58	0.34–7.40	
	T3	0.00	0.00		1.02	0.11–9.96		3.22	0.20–52.13		1.44	0.13–15.93	
	T4	10.53	1.32–83.90		8.49	2.45–29.34		10.55	1.32–84.19		12.59	2.82–56.04	
N stage	0	1		0.387	1		0.079	1		0.397	1		0.094
	1	4.00	0.81–19.88		6.52	1.37–30.99		4.51	0.49–41.69		11.60	1.39–97.01	
	2	2.58	0.51–13.03		6.85	1.52–30.78		5.36	0.62–46.59		13.83	1.79–108.74	
	3	3.26	0.44–24.39		3.99	0.54–29.80		8.72	0.42–107.25		9.11	0.37–105.87	

LV-IMRT, large-target-volume intensity-modulated radiotherapy; RV-IMRT, reduced-target-volume intensity-modulated radiotherapy; OS, overall survival; PFS, progression-free survival; DMFS, distant metastasis-free survival; LRFFS, locoregional recurrence-free survival; RR, relative risk; CI, confidence interval;

*, Statistical significance.

### Toxicity profiles

Table 5 shows the radiation toxicity profiles for both groups. Overall, 31.3% of the LV-IMRT patients and 25.5% of the RV-IMRT patients suffered from acute Grade 3/4 radiation mucositis. Furthermore, 25% of patients in the LV-IMRT group and 25.5% of the patients in the RV-IMRT group presented with acute Grade 3 radiation dermatitis. There were no statistically significant differences in the rates of acute radiation dermatitis and mucositis between the two groups. However, significantly fewer cases of Grade 2 and 3 delayed-toxicity xerostomia were noted in the RV-IMRT group than in the LV-IMRT group (P < 0.001; Grade 2, LV-IMRT vs. RV-IMRT, 89.5% vs. 56.4%; Grade 3, LV-IMRT vs. RV-IMRT, 4.2% vs. 0%).

**Table 5:** Radiation toxicity profiles.

	LV-IMRT	RV-IMRT	Sig.
Radiation mucositis (%)			
Grade 0	0.0	3.6	0.476
Grade 1	10.4	14.5	
Grade 2	58.3	56.4	
Grade 3	27.1	25.5	
Grade 4	4.2	0.0	
Radiation dermatitis (%)			1.000
Grade 0	2.1	3.6%	
Grade 1	14.6	14.5%	
Grade 2	58.3	56.4%	
Grade 3	25.0	25.5%	
Xerostomia (%)			<0.001*
Grade 0	0.0	5.5	
Grade 1	6.3	38.1	
Grade 2	89.5	56.4	
Grade 3	4.2	0.0	

LV-IMRT, large-target-volume intensity-modulated radiotherapy; RV-IMRT, reduced-target-volume intensity-modulated radiotherapy; Sig., significance

*, Statistical significance.

### Salvage treatment

Of the 27-locoregional recurrence events, only 8 patients had successful salvage treatments. One regional failure was rescued using radical neck dissection. In 7 cases, recurrence at the primary site was rescued using concurrent chemoradiotherapy, stereotactic body radiation therapy, or IMRT alone. Three patients with distant metastasis (one lung metastasis, one liver metastasis, and one multiple metastases) were rescued by radical surgical intervention and chemotherapy.

## Discussion

Beginning in the early 1970s, conventional radiotherapy was used to treat NPC by delivering radical radiation doses (60–70 Gy) using two laterally opposing fields to all areas potentially harboring gross or microscopic disease tissue, as well as to anatomical structures that were at risk for tumor invasion in the vicinity of the nasopharynx[16]. In recent decades, IMRT has largely replaced 2-D field-base radiotherapy for the treatment of NPC, and the overall local control rate for NPC has been improved from 80%[17, 18] to 90% with the adoption of 3DCRT or IMRT[1–4, 6, 7, 19].

The survival rate of NPC patients has been prolonged with IMRT and chemotherapy, there has been growing concern regarding late toxicity effects induced by radiotherapy and long-term quality of life for NPC survivors. As a result, target-oriented techniques and selective neck irradiation are increasingly used for the treatment of NPC. Although there has been some concern[20] regarding such target-orientated and reduced-volume techniques, recent prospective studies[19, 21] have demonstrated the efficacy and feasibility of these approaches.

Although several groups have published atlases for defining CTVs for elective lymph-node treatment of head-and-neck cancer[22], no general consensus exists as how to extend the GTV to the CTV. In general, protocols for extending the GTV to the CTV were developed using the concept of subclinical disease spreading and the field borders used in conventional radiotherapy; however, different institutions often have different methods for defining and expanding GTV margins to create CTVs. Expansion of the GTV to the CTV70 for NPC treatment is commonly carried out through geometric expansion. Previously published institutional protocols[1, 2, 4, 5] call for expanding the GTV 2–10 mm to create the CTV70 and another 3–5 mm to create the PTV70, yielding a total expansion range of 5–15 mm. Recent RTOG trials involving IMRT of NPC[6, 7] suggests using a 5-10-mm margin for expanding the GTV to the PTV70, and in areas where the GTV or CTV is adjacent to critical structures, they suggest that the margin can be reduced to 1 mm. In other words, the current diversity in GTV to CTV margin definitions reveals an underlying uncertainty concerning the true subclinical extension range of the gross tumor in NPC.

The rapid development of technologies in both radiological imaging and radiation therapy over the past decades has improved the accuracy of gross tumor delineation[23, 24], to the point where margin expansion from the GTV to the CTV could be omitted[25]. The rationale for reducing the volume of IMRT is to limit the region exposed to radical radiation doses to only the area containing the gross tumor, while at the same time minimizing unnecessary radiation doses to normal tissue. Indeed, if subclinical cancer cells deposit outside the imaging-based GTV, the relatively high radiation doses (60-70Gy) surrounding the GTV should be sufficient for treating the subclinical disease[26]. Caudell et al.[10] reported that there were no differences in local control between head- and- neck cancer patients treated using GTV to PTV expansions of 4–6 mm and patients treated with expansions of 10–15 mm or even >15 mm. With respect to their in-field failures, a median GTV volumetric expansion of 6mm (range, 0-15mm) was required to contain the contoured recurrence structures. For a case of marginal failure, a 3.3-cm expansion was required to encompass the entire recurrence area, suggesting that these lesions would have been unresponsive to radiotherapy regardless of margin expansion. Similarly, the results shown here suggest that the high-dose treatment volume may only require a 5 mm margin expansion.

In this study, we carried out a retrospective analysis of RV-IMRT by reducing the GTV to the CTV70 margin (from 7 mm to 0 mm), the CTV70 to the PTV70 margin (from 7mm to 5 mm), and total extension of the GTV to the PTV (14 mm to 5 mm). For the RV-IMRT group, the high LRFS (83.5%), RRFS (92.6%), and OS (91.0%) rates were in agreement with previous NPC studies[1–4, 6, 7]. The RV-IMRT technique did not alter the NPC treatment failure pattern either. Indeed, failures due to locally advanced disease and distant metastasis remain the primary problems for treating NPC. The majority of locoregional failures, which were observed at a rate of 77.7% in the current study, were within the high-dose region (the median dose of the in-field failures was 70Gy). Failures within and around the high-dose region should be considered as true failures with no apparent technical cause[4]. Furthermore, the RV-IMRT group did not show any marginal or out-field failures. Surprisingly, the overall survival for the RV-IMRT group is significant better than the LV-IMRT group. The reason could be multifactorial. Since the interval for enrollment was longer than 5 years, various factors such as the image-guidance radiotherapy, the quality of image, and regimens of salvage treatment have evolved.

More advanced T stage is associated with pooler local control and higher rate of distant metastasis, and T4 disease remains a challenge for NPC therapy[27, 28]. 56.3% of the local failure cases in our study cohort were due to T4 disease. The T4 failure rate was 25% in the RV-IMRT group (4 T4 patients, 1 recurrence) versus 57% in the LV-IMRT group (14 T4 patients, 8 recurrences). However, critical neurological organs, such as the brain stem, optic apparatus, temporal lobe, and spinal cord, remaining limiting factors for the delivery of high-dose radiation to T4 disease patients.

As outcomes improve and survival times lengthen, the prevention of radiation-induced complications is becoming increasingly important, and a number of studies have demonstrated quality-of-life improvements from IMRT[29–31]. Although RV-IMRT increased dose conformity within the treatment targets and reduced the mean volume of tissue receiving 70Gy by 46%, comparing to the LV-IMRT groups, we did not observe a significant reduction in acute radiation-induced toxicity. The percentages of Grade 3/4 mucositis and dermatitis cases were not significantly different between the two groups. Therefore, dosimetric improvements did not always translate into absolute improvements in toxicity scores. Consistent with our findings, Anne Lee et al.[4] reported similar acute toxicity results for NPC treated with IMRT. However, we did note during our recent clinical observation that both the duration and extent of radiation-induced dermatitis and mucositis were shorter and smaller, respectively, in the RV-IMRT group compared to the LV-IMRT group, which was not easy to determine from the CTCAE scoring system.

Sparing of the parotid gland was not our first goal, as many gross neck lymph-node metastases are located in the retropharyngeal and level II regions. Additionally, it can be difficult to avoid the parotid gland and still maintain adequate lymph node coverage with a high radiation dose. In this study, we did not observe any failures around the parotid regions. Furthermore, RV-IMRT reduced—by a small but significant amount—the dose of radiation delivered to the right parotid glands, which reduced the severity of late xerostomia. These findings are in agreement with a recent study by Vergeer et al.[32], which showed that after 6 months, the prevalence of patient-rated moderate-to-severe xerostomia and Grade 2 or higher RTOG xerostomia was significantly lower following IMRT than after 3D-conformal radiotherapy.

Our study has several limitations. First, it is a retrospective study with a small amount of patients in the each group. Second, not complete assessment of all late-side toxicities and quality of life, which could the important reasons why the RV-IMRT group has a better overall survival rate. Third, due to the relatively small sample size in the both groups, the current findings could only be taken as preliminary. Therefore, well-designed prospective trials and long-term follow-up are needed for further research.

## Conclusions

By incorporating improvements in modern IMRT and imaging technologies with a better understanding of tumor biology, we were able to deliver radical-radiation doses to more precise target areas without compromising NPC treatment. In this retrospective study, we observed no increased risk of disease failure using reduced-volume, high-dose CTVs and PTVs when treating NPC patients with IMRT. Furthermore, RV-IMRT led to reduced delayed-toxicity xerostomia compared to LV-IMRT, leading to improved patient quality of life.
